# Analysis of salivary detection of P16INK4A and RASSF1A promoter gene methylation and its association with oral squamous cell carcinoma in a Colombian population

**DOI:** 10.4317/jced.56647

**Published:** 2020-05-01

**Authors:** Leonor-Victoria González-Pérez, Diana-María Isaza-Guzmán, Eduin-Alonso Arango-Pérez, Sergio-Iván Tobón-Arroyave

**Affiliations:** 1 Associate Professor. Laboratory of Histopathology, Faculty of Dentistry, University of Antioquia. Medellín, Colombia; 2 Titular Professor. Laboratory of Immunodetection and Bioanalysis, Faculty of Dentistry, University of Antioquia. Medellín, Colombia; 3 Oral and Maxillofacial Surgeon Resident. Department of Oral and Maxillofacial Surgery, Faculty of Dentistry, University of Antioquia. Medellín, Colombia; 4 Stomatologist and Oral Surgeon. Stomatology and Maxillofacial Surgery Unit, San Vicente Foundation University Hospital, Medellín, Colombia

## Abstract

**Background:**

Epigenetic factors play a fundamental role in the etiopathogenesis of oral squamous cell carcinoma (OSCC). This study evaluated if salivary detection of P16INK4A/RASSF1A gene promoter methylation might be linked to the clinical/histological features of OSCC in a Colombian population.

**Material and Methods:**

Methylation-specific polymerase chain reaction (MSP-PCR) was used to detect the methylation frequency of P16INK4A/RASSF1A genes in DNA obtained from whole saliva collected of 40 healthy controls (HC) and 43 OSCC patients. Determination of the clinical performance of MSP-PCR assay was based on standard algorithms derived from two-way contingency table analysis. The association of methylation status of targeted genes with OSCC was analyzed in a multivariate binary logistic regression model.

**Results:**

There were significantly higher proportions of promoter methylation of these target genes in OSCC patients when compared with HC. The analysis of single methylated genes showed high specificity, good positive and negative predictive values, but was accompanied by a low sensitivity. OSCC cases with clinical stage III/IV, poorly differentiated, and severe cellular atypia showed a significantly greater proportion of methylated than that of unmethylated targeted genes in saliva samples. Logistic regression analysis indicated an independent association of P16INK4A and RASSF1A promoter methylation with OSCC diagnosis. A significant interaction effect between ageing and P16INK4A promoter methylation was also detected.

**Conclusions:**

Salivary detection of P16INK4A and RASSF1A promoter methylation appears to be independently associated with OSCC and may be linked to the tumor activity in the present population. Consequently, the targeting of these genes in saliva samples might constitute an important tool for diagnosis and prognosis purposes.

** Key words:**Gene methylation, oral squamous cell carcinoma, P16INK4A, RASSF1A, saliva.

## Introduction

Oral squamous cell carcinoma (OSCC) is defined as a malignant epithelial tumor showing squamous differentiation characterized by the formation of keratin pearls and/or intercellular bridges (1). It has been acknowledged that environmental stimuli, including alcohol consumption, smoking, chewing betel nuts, and ultraviolet radiation may modulate a chronic, complex, multistep, and sequential process of oral carcinogenesis (2) which finally results in abnormalities of cell growth regulation and differentiation (1,3). Notwithstanding, the key mediators that influence on this process have been not yet fully clarified. Increasing evidence suggests that malignant transformation in the oral mucosa can arise as a result of mutations in the genes that act in the regulation of cell growth and in the processes of DNA repair (4), thus inducing cell proliferation, abnormal keratinization, epithelial dysplasia, cellular mobility, and angiogenesis (5). Likewise, several epigenetic mechanisms such as methylation, may alter gene expression leading to cellular functional alterations that causes abnormal division patterns or cell death (6-9).

The methylation phenomenon of the *P16INK4A* and *RASSF1A* tumor suppressor genes (TSGs), involved in the cell cycle control, may lead to the silencing of these genes, thus favoring the abnormal proliferation of the involved cells and tumorigenesis (10-12). The methylation process includes the transfer of a methyl group to carbon 5 of cytosine in CpG dinucleotide-rich regions named CpG islands (13). The *P16INK4A* gene product is a negative regulator of the cell cycle that inhibits cyclin-dependent kinases 4 and 6 and causing the blockade of cell cycle progression from G1 to S phases (10-12). The *RASSF1A* gene, in turn, plays a fundamental role in the cell cycle control by constraining the accumulation of cyclin D1 and inducing cell cycle arrest (14).

It has been postulated that the analysis of DNA methylation in body fluids such as saliva possess great diagnostic accuracy for timely detection of head and neck squamous cell carcinoma (HNSCC) (7,15). Since not only OSCC is dissimilar from cancers of the other sites of the head and neck area (12,16), but also that epigenetic alterations can differ depending of ethnic populations or geographic areas (17,18), this study intended to investigate whether the salivary detection of *P16INK4A* and *RASSF1A* promoter methylation might be linked with the clinical and histological features of OSCC in a Colombian population.

## Material and Methods

-Study design

**Figure 1:**
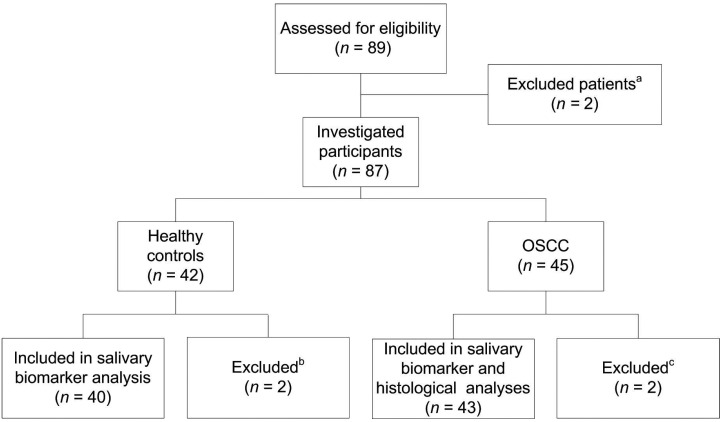
Flow chart illustrating the progress of participants through the study. Patients were excluded because: (a) they did not give consent or did not return the study forms; (b) saliva samples were not provided by the participants; (c) they declined to participate or have incomplete medical records.

This cross-sectional analytic study was conducted following the ethical guidelines of the Helsinki Declaration and ethical approval was obtained from the Institutional Ethics Committees for Human Studies of the University of Antioquia (Concept Number 16-2016) and San Vicente Foundation University Hospital (Reference number 1482-2016). Data are presented following the guidelines of the Strengthening the Reporting of Observational Studies in Epidemiology (STROBE) statement for case-control studies. The original cohort comprised 89 volunteers evaluated between January 2017 and July 2019 in the Stomatology and Maxillofacial Surgery Unit of the University Hospital San Vicente Foundation in Medellín (Colombia). Based on this cohort, and considering a distribution of cases and controls of 50%, power calculation using a web-based statistical sample size calculator (Raosoft® Inc., Seattle, WA, USA), generated a sample size requirement of at least 68 participants to achieve a 90% confidence level, with an alpha value of 5% and a power >84% in identifying significant differences in the detection rate of methylation status. However, a major number of participants were recruited to improve the statistical power and precision. Fig. 1 depicts the distribution of this initial cohort leading to the definite inclusion of 83 participants. Once the volunteers signed the informed consent to be included in the study, they were interviewed using a standardized questionnaire with the purpose of collecting demographic and medical information such as gender, age, smoking habit, and alcohol consumption.

The study population included a group of 43 patients with untreated OSCC and a group of 40 healthy controls (HC) with no evidence of oral or systemic disease. The exclusion criteria defined were individuals that refused to give informed consent or not provide saliva/tissue samples, patients previously irradiated in head and neck region, cases of metastatic lesions of unknown origin, history of oral potentially malignant disorders, and incomplete medical records. An oral clinical examination was performed in all participants synchronously by two trained researchers (H.D. M-D. and E.A. A-P.) to discard the presence of pathological conditions of the oral mucosa that could affect the results. The clinical information that was gathered for OSCC group included the lesion size and location. Subsequently, these cases were subjected to clinical and tomographic staging according to previously defined criteria (19). At least two tissue specimens, ranging from 5 to 10 mm in size, were taken from different areas of patients with OSCC diagnosis by incisional biopsy achieved under regional anesthesia in an operating room by the same researchers that accomplished the data collection and clinical examination (H.D. M-D. and E.A. A-P.). Histological assessment was made on 4 µm-thick haematoxylin-eosin stained sections. Ten high-power non-overlapping fields (400X) were examined for each tumor by a trained and calibrated researcher (L.V. G-P.) with a Zeiss Axiolab® light microscope (Carl Zeiss®, Oberkochen, Germany) equipped with an image analyzer system (Zen Pro®, Carl Zeiss®). Histological assessment considered the degree of invasion, presence/absence of perineural/lymphovascular invasion, histologic grade, cellular atypia, inflammatory infiltrate density, and average of mitotic/apoptotic Figures of the tumors.

Each participant provided a sample of 5 mL of unstimulated whole saliva by spitting into 50 mL sterile centrifuge tubes. The samples were centrifuged at 800 x g for 10-min and the pellet was removed to be dispersed using a vortex for 15-sec and stored at -20 °C until processed. For DNA extraction, 200 µL of each pellet were processed with the QIAamp® DNA blood mini kit (Qiagen Sciences®, GmbH, Hilden, Germany) following the spin protocol for DNA purification from body fluids according to manufacturer’s instructions. DNA was stored frozen at -20ºC until methylation-specific polymerase chain reaction (MSP-PCR) was performed.

**Figure 2:**
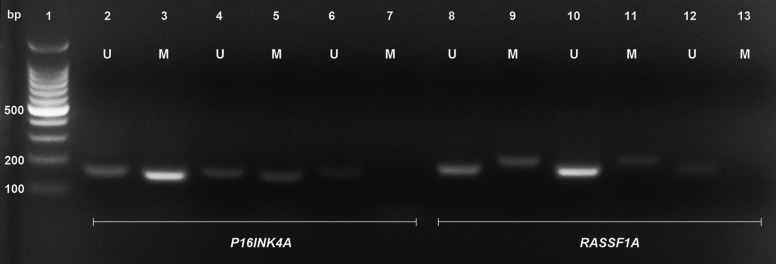
Representative image of a 2% (w/v) ethidium bromide-stained agarose gel showing results of MSP-PCR assay of the promoter hypermethylation events for *P16INK4A* and *RASSF1A* TSGs. M indicates the presence of methylated and U indicates the presence of unmethylated *P16INK4A* and *RASSF1A* TSGs. Lane 1, 100 bp DNA ladder. Lanes 2 to 7 correspond to *P16INK4A* analysis as follows: lane 2, unmethylated human control DNA; lane 3, methylated human control DNA; lanes 4 to 7, DNA from patients; Lanes 8 to 13 correspond to *RASSF1A* analysis as follows: lane 8, unmethylated human control DNA; lane 9, methylated human control DNA; lanes 10 to 13, DNA from patients.

Each genomic DNA sample was treated with sodium bisulfite for conversion of unmethylated cytosines residues into uracil, leaving unaffected the methylated cytosines using the Epitect® Plus DNA Bisulfite Kit (Qiagen Sciences®) according to the manufacturer’s guidelines. The analysis of *P16INK4A* and *RASSF1A* promoter methylation was performed according to established protocols (8,20). For interpretation of results, the presence of any detecTable amplification product using a methylated allele-specific primer set was considered as positive (Fig. 2). Specific primers used for determining the methylation status of the *P16INK4A* and *RASSF1A* TSGs are listed in Table 1.

**Table 1:**
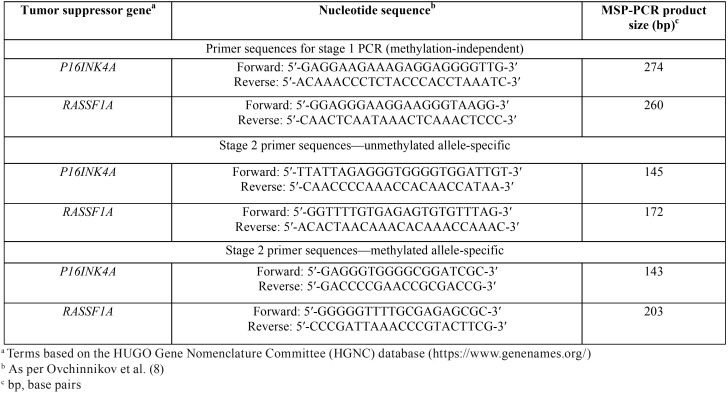
Description of the MSP-PCR primers used for analyzing methylation status of the *P16INK4A* and *RASSF1A* TSGs.

-Statistical analysis

Data collected were analyzed using the SPSS 25.0® (IBM, Armonk, NY) statistical package. Reproducibility analysis for MSP-PCR assays was performed processing again the saliva samples obtained from 6 participants randomly selected using the Epidat 4.0® (PAHO/WHO, Washington, DC, USA) software. The interval amongst repeated tests was six months. The concordance between the two series of data was quantified using the Cohen’s kappa statistic (κ). Afterward, bivariate comparisons were performed Fisher exact test or Pearson’s chi-square (χ2) test in order to examine potential between-group differences regarding demographic, clinicopathological, and molecular findings and to identify potential explanatory and/or confounding variables for association with OSCC. Also, determination of the clinical performance of MSP-PCR assay in relation to diagnosis was performed using an online calculator (http://StatPages.info/ctab2x2.html). Subsequently, univariate and multivariate binary logistic regression analyses were conducted to evaluate the association of genetic variables regarding OSCC whilst adjusting for demographic variables with P-value ≤0.20 identified in the bivariate analysis. Positive associations existed when the OR was >2 and the confidence range did not include 1.0. *P* values <0.05 were considered statistically significant.

## Results

-Demographic and behavioral profile of the study population

The results of evaluation of demographic and behavioral parameters in the study population are displayed in Table 2. From this Table is evident that although no significant differences (*P* >0.05, χ2 test) between clinical groups regarding gender were detected, the age stratum ≥50 years, current/former smokers, and current/former alcohol drinkers were significantly more frequent (all *P* <0.05, χ2 test) in OSCC group in comparison with HC. Consequently, these three later variables fulfil the conditions to be considered confounders of the association among MSP-PCR findings and disease status.

**Table 2:**
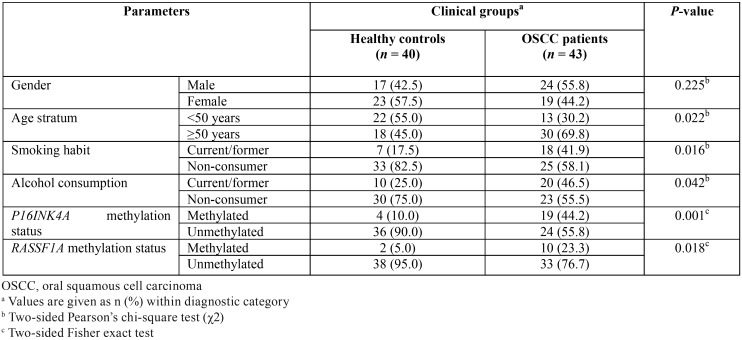
Bivariate comparisons of demographic parameters and gene promoter methylation status from the study population according to diagnosis category.

-*P16INK4A* and *RASSF1A* methylation status

The intra-assay reproducibility was excellent for both *P16INK4A* and *RASSF1A* gene promoter methylation status (ĸ = 1.000). The results of estimation of methylation status of *P16INK4A* and *RASSF1A* TSGs are also presented in Table 2. As can be seen, promoter methylation of the two targeted genes was significantly more frequent in OSCC patients when compared with HC (all *P* <0.05, Fisher exact test). Likewise, there was a significant higher proportion (*P* <0.001, χ2 test, data not shown) of OSCC patients (23 cases, 53.5%) presenting at least one methylated marker in saliva in comparison with HC (5 cases, 12.5%). Conversely, although there was a greater number of OSCC patients presenting two target genes methylated (5 cases, 11.6%) when compared with HC (1 case, 2.5%), this difference was not statistically significant (*P* >0.05, Fisher exact test, data not shown). In addition, promoter methylation was further assessed regarding significant demographic and behavioral variables, irrespective of diagnosis group, in order to determine variations that might potentially affect the results. Accordingly, no statistical differences were observed among age stratum, smoking habit, and alcohol consumption subgroups regarding the frequency of *P16INK4A* and *RASSF1A* promoter methylation (all *P* values >0.05, Fisher exact and χ2 tests; data not shown), thus suggesting an optimal comparability of the data between cases and controls.

The analysis of the clinical performance of methylation status showed sensitivity values relatively low for detecting methylation of *P16INK4A* (44.2%) and *RASSF1A* (23.3%) when analyzed individually in saliva samples. On the contrary, the test yielded high specificity for both *P16INK4A* (90.0%) and *RASSF1A* (95.0%) methylation. Moreover, this test also revealed relatively high positive predictive values (PPV) for *P16INK4A* (82.6%) and *RASSF1A* (83.3%) methylation and good negative predictive values (NPV) for *P16INK4A* (60.0%) and *RASSF1A* (53.5%) methylation. Notwithstanding, it was remarkable that the combined analysis of the two genes was able to provide a sensitivity of methylation detection of 53.5%, with a specificity of 87.5%, PPV of 82.1%, and NPV of 63.6%.

-Comparison between promoter methylation status and clinicopathological characteristics of OSCC patients

Bivariate comparisons among promoter methylation status of each gene with the clinical and histological characteristics of the patients with diagnosis of OSCC are depicted in Table 3. As can be observed, whereas no significant differences (*P* >0.05, Fisher exact and χ2 tests) were apparent in the methylation frequency of *P16INK4A* and *RASSF1A* genes with respect to location of the lesions, degree of invasion, perineural/lymphovascular invasion, inflammatory infiltrate density, nor apoptotic/mitotic counts, those OSCC cases with clinical stage III/IV, poorly differentiated, and severe cellular atypia showed a significantly greater proportion (*P* <0.05) of methylated than unmethylated targeted genes in saliva samples.

**Table 3:**
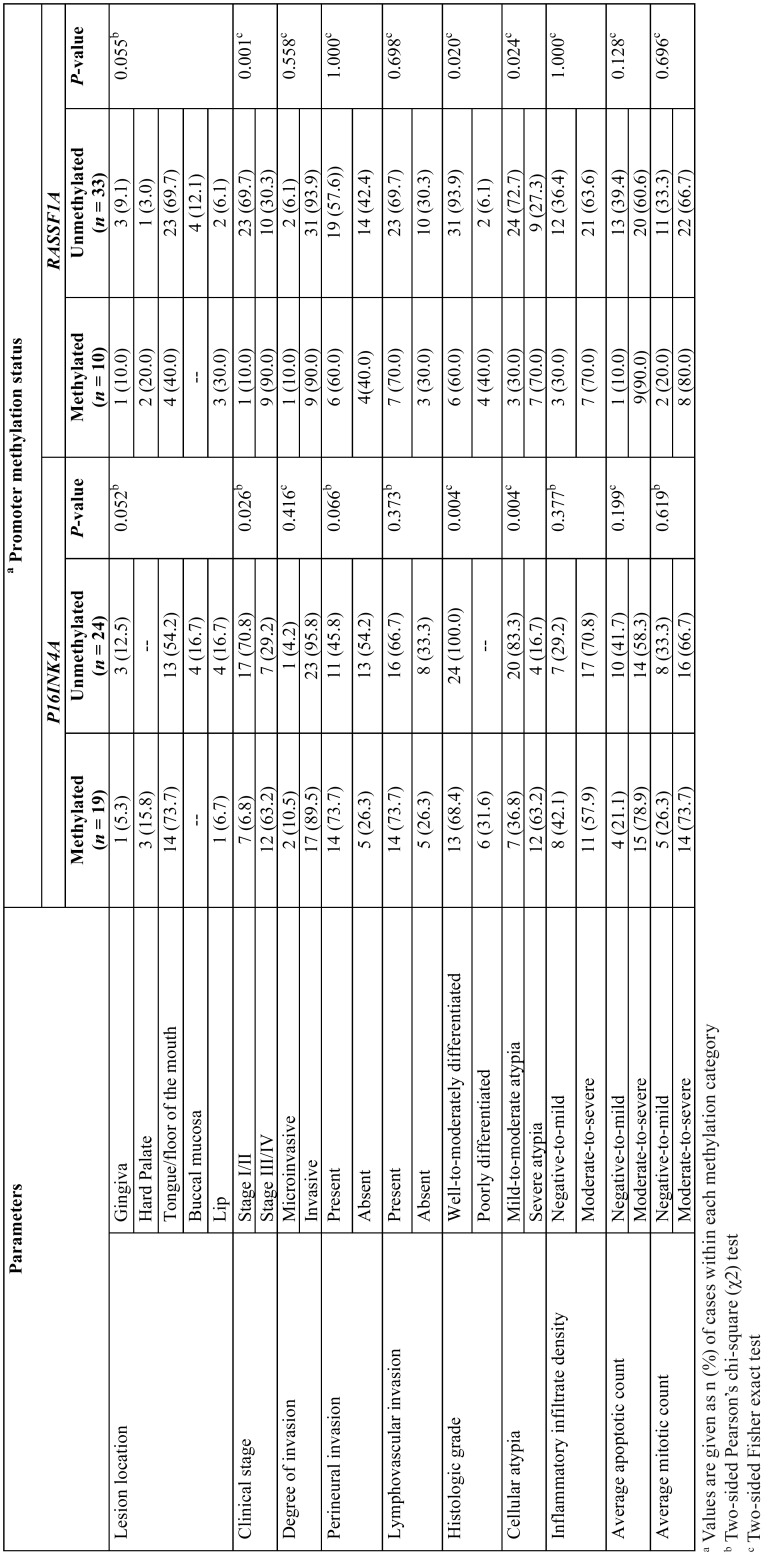
Bivariate comparisons among clinical and histological variables regarding promoter methylation status of *P16INK4A* and *RASSF1A* TSGs in oral squamous cell carcinoma patients.

-Results from multivariate binary logistic regression analysis

Table 4 illustrates the results from multivariate binary logistic regression models for the association of *P16INK4A* and *RASSF1A* methylation status with OSCC after adjusting for age stratum, smoking habit, and alcohol consumption. The Hosmer-Lemeshow test values showed that the logistic models fit the data moderately well (*P* >0.05). Equally, c-statistic values closer to 1.0 for the two TSGs indicated a great discriminative power. It may be also appreciated that the OR for OSCC was significantly increased (*P* <0.05, Wald’s test) for patients with methylated *P16INK4A* and *RASSF1A* TSGs. After adjusting for demographic/behavioral confounders identified in the bivariate analyses, the two biomarkers remained independently associated with OSCC (*P* <0.05, Wald’s test). Additionally, a significant synergistic biological interaction (adjusted OR, 9.17; 95% CI = 1.93 to 43.59; *P* = 0.005, Wald test) could be noted between *P16INK4A* promoter methylation and age ≥50 years in patients with OSCC.

**Table 4:**
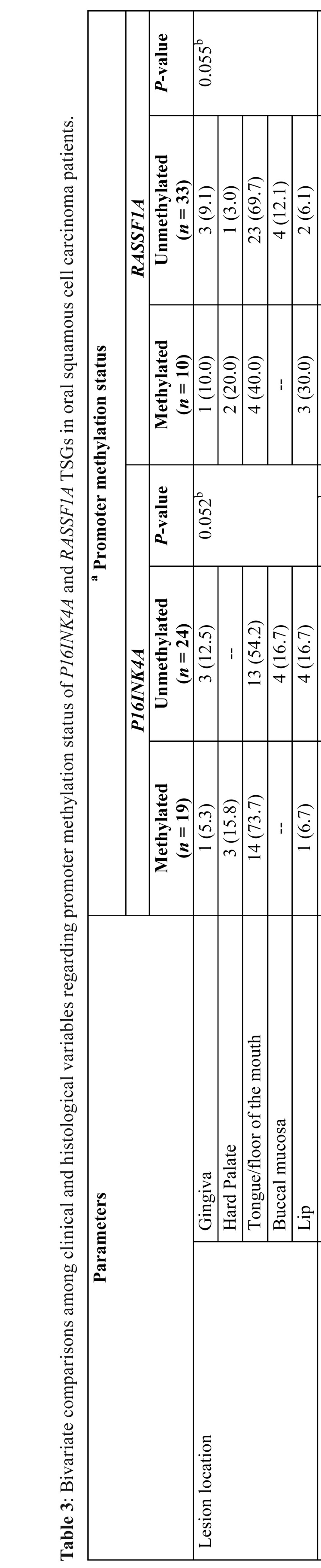
Summary of univariate and multivariate binary logistic regression analyses for the association of promoter methylation status of *P16INK4A* and *RASSF1A* TSGs with oral squamous cell carcinoma adjusting for age, smoking habit, and alcohol consumption.

## Discussion

Although several studies have been performed to assess the association of *P16INK4A* and *RASSF1A* promoter methylation regarding to HNSCC risk in saliva samples (7,8,15), to date, only two studies have focused specifically on their role in OSCC (17,21). Considering that it has been found evidence of substantial molecular diversity between different HNSCCs (22), it would be possible to assume that methylation frequency of these two TSGs related to OSCC might differ with that of other locations of the head and neck region. Moreover, as differences in molecular characteristics of OSCC associated to geographic location have been found (17,18), the current study investigated whether if methylation status of *P16INK4A* and *RASSF1A* TSGs, as detected in saliva samples, might be linked with OSCC in a group of Colombian individuals. Therefore, this study comprised a representative sample of patients with diverse clinical stages across the whole spectrum of disease evolution. Also, although cases and controls groups were relatively homogeneous regarding gender, parameters such as age, smoking habit, and alcohol consumption disclosed significant between-group differences, and fulfil the preset conditions to be selected as confounders for the association between MSP-PCR findings and OSCC in the final models.

It has been recognized that DNA methylation in TSGs is a common event in human neoplasms (23). In consistency with the former, this study showed that the percentage of positive samples of promoter methylation for each TSG was significantly higher in individuals with OSCC when compared with HC. Additionally, 53.5% of cases showed methylation of at least one gene and 11.6% had both *P16INK4A* and *RASSF1A* gene promoters methylated. In contrast, the simultaneous methylation of *P16INK4A* and *RASSF1A* gene promoters was only observed in one of the controls. This is of importance because might corroborate the concept that DNA methylation plays a major role in OSCC development (21). Nevertheless, the results of methylation detection frequency for targeted TSGs have remained inconsistent amongst the studies given the high variability of frequencies reported. In the present study, the detection frequency of *P16INK4A* promoter methylation was 44.2%, while other studies using saliva as a source of genomic DNA show detection rates of 47.8% (17) and 17.2% (21) in OSCC, and of 47.0% (24), 29.0% (15), and 25.0% (8), in HNSCC. Alternatively, *RASSF1A* promoter methylation was positive in 23.3% of the patients, closely equivalent to what was detected in Europeans (20.0%) (15), but lower that what reported in saliva obtained from HNSCC patients in Australians (50.0%) (8). Although the present findings can have been influenced by ethnic factors and might reflect a geographic variation, other reasons for differences may be attribuTable to variations in sample processing and assay protocols, clinical stage of the tumors, and environmental stressors.

The available evidence suggests that diagnostic accuracy of DNA methylation for HNSCCs varies according the sample type and number of markers tested (7,25). According to the present data, the analysis of single methylated genes showed high specificity, high PPV, and good NPV, but was accompanied by a low sensitivity. Even so, the combined analysis of the two genes led to improve the sensitivity to 53.5% maintaining a specificity of 87.5%, which concurs with the theory that DNA methylation detected in saliva samples had an overall sensitivity and specificity for HNSCC diagnosis of 47% and 89%, respectively (25). At the same time, and since the combined PPV and NPV were 82.1% and 63.6% respectively, it would be feasible to state that combined salivary analysis of *P16INK4A* and *RASSF1A* gene methylation was reasonably discriminatory for OSCC detection in this study population.

Alongside the *P16INK4A* and *RASSF1A* promoter methylation detection, several investigations have intended to determine an association with clinicopathologic features. However, the results have been inconsistent, since while some studies have not found clear evidence of significant associations with clinicopathologic features (2,6,10), others have demonstrated that methylation of these TSGs is significantly associated with lymph node metastasis (15,17), clinical stage (6,8,26), and histologic grade (8,15,26). In the present study, patients with stage III/IV tumors showed a significantly greater proportion of methylated than that of unmethylated targeted genes in saliva samples, thus suggesting that although the imbalance of DNA methylation may be an early event in the carcinogenic pathway of OSCC (12), its detection may increase with the advancement of the tumor stage (8). Alternatively, those lesions exhibiting severe cellular atypia and poor histologic differentiation showed a significantly greater percentage of methylated than that of unmethylated genes in saliva samples. Taking into account not only that atypia is associated with loss of tight junction adhesion (27), but also that aggressive tumors may undergo increased rate of mechanical dissociation or shedding into salivary secretions (7), it would be possible to hypothesize that *P16INK4A/RASSF1A* methylation may be associated with high cell proliferation yielding a high concentration of the methylated signal in exfoliated cells. In concordance with the former, it has been previously stated that salivary detection of DNA tumor-suppressor methylation genes might mirror actual tumor activities and that could be used as a marker for disease progression (28).

Some researchers have established that DNA methylation may be affected by demographic variations such as gender, ageing, and smoking/alcohol consumption (12,18). Though the present findings revealed an independent association of *P16INK4A* and *RASSF1A* gene methylation with disease status, a synergistic biological interactive effect between salivary *P16INK4A* gene methylation and ageing was also present in the OSCC group. This finding may suggest that ageing-deleterious effects of oral epithelial cells could be important modifying factors for the effect of *P16INK4A* gene methylation in OSCC. In accordance with the former, synergistic patterns between ageing and DNA methylation have been previously acknowledged (29,30). Although it is difficult to establish the exact role of age-related changes in oral carcinogenesis, it has been stated that almost one third of the CpG sites reveal ageing-associated DNA methylation changes and that genes that are mandatory for the differentiation of epithelial cells are more likely to become methylated with increasing age (30).

To finalize, two limitations were evident in this research. First, the cross-sectional nature of this study hinders the assessment of a causal relationship between methylation of specific TSGs regarding the progression or recurrence of OSCC. Hence, prospective longitudinal approaches are required to study and establish these relationships. Second, the small sample size in association with the variability of clinicopathological patterns of disease between the study participants and selected patient population might have influenced the results. Therefore, replication in further studies with larger sample sizes and with other ethnicities is essential to confirm the generalizability of present findings. Even so, taking into account that the rule of thumb for stability of the estimates from logistic regression is to have at least 10-20 events per predictor used in the model, and given that there were 19 and 10 methylated cases within the OSCC category for *P16INK4A* and *RASSF1A* in the adjusted model respectively, it would be possible to assume that the results are relatively sTable.

In summary, the findings of this study suggest that salivary detection of *P16INK4A* and *RASSF1A* promoter methylation appears to be independently associated with OSCC and may be linked to the tumor activity in the present population. Consequently, the targeting of these genes in saliva samples might constitute an important tool for diagnosis and prognosis purposes.
